# Factors Promoting Lipopolysaccharide Uptake by Synthetic Lipid Droplets

**DOI:** 10.1021/acsomega.4c09599

**Published:** 2025-02-10

**Authors:** Assame Arnob, Anirudh Gairola, Hannah Clayton, Arul Jayaraman, Hung-Jen Wu

**Affiliations:** † Artie McFerrin Department of Chemical Engineering, Texas A&M University, College Station, Texas 77843, United States; ‡ Department of Biomedical Engineering, Texas A&M University, College Station, Texas 77843, United States

## Abstract

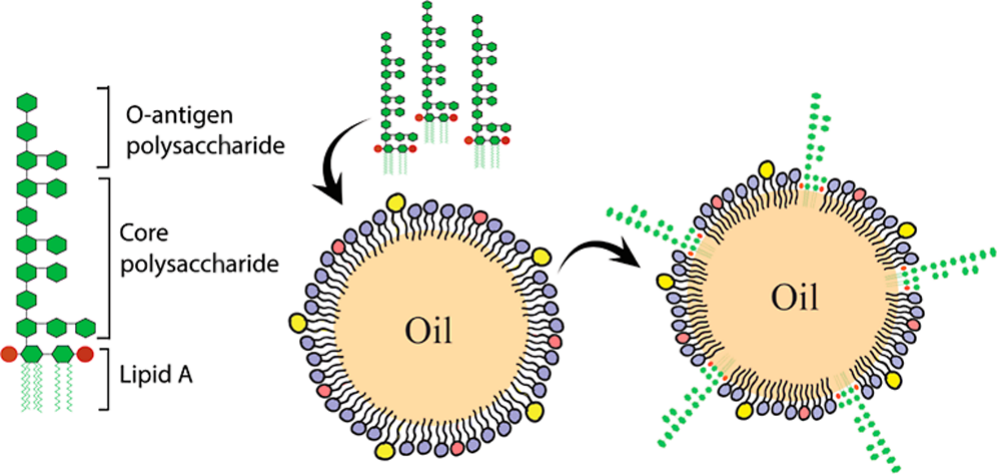

Lipoproteins are essential in removing lipopolysaccharides (LPSs) from blood during bacterial inflammation. The physicochemical properties of lipoproteins and environmental factors can impact LPS uptake. This work prepared synthetic lipid droplets containing triglycerides, cholesterols, and phospholipids to mimic lipoproteins. The physicochemical properties of these lipid droplets, such as charges, sizes, and lipid compositions, were altered to understand the underlying factors affecting LPS uptake. The amphiphilic LPS could spontaneously adsorb on the surface of lipid droplets without lipopolysaccharide-binding protein (LBP); however, the presence of LBP can increase the LPS uptake. The positively charged lipid droplets also enhance the uptake of negatively charged LPS. Most interestingly, the LPS uptake highly depends on the concentrations of Ca^2+^ near the physiological conditions, but the impact of Mg^2+^ ions was insignificant. The increase in Ca^2+^ ions can improve LPS uptake by lipid droplets; this result suggested that Ca^2+^ may play an essential role in LPS clearance. Since septic shock patients typically suffer from hypocalcemia and low levels of lipoproteins, the supplementation of Ca^2+^ ions along with synthetic lipoproteins may be a potential treatment for severe sepsis.

## 1 Introduction

**Figure 1:**
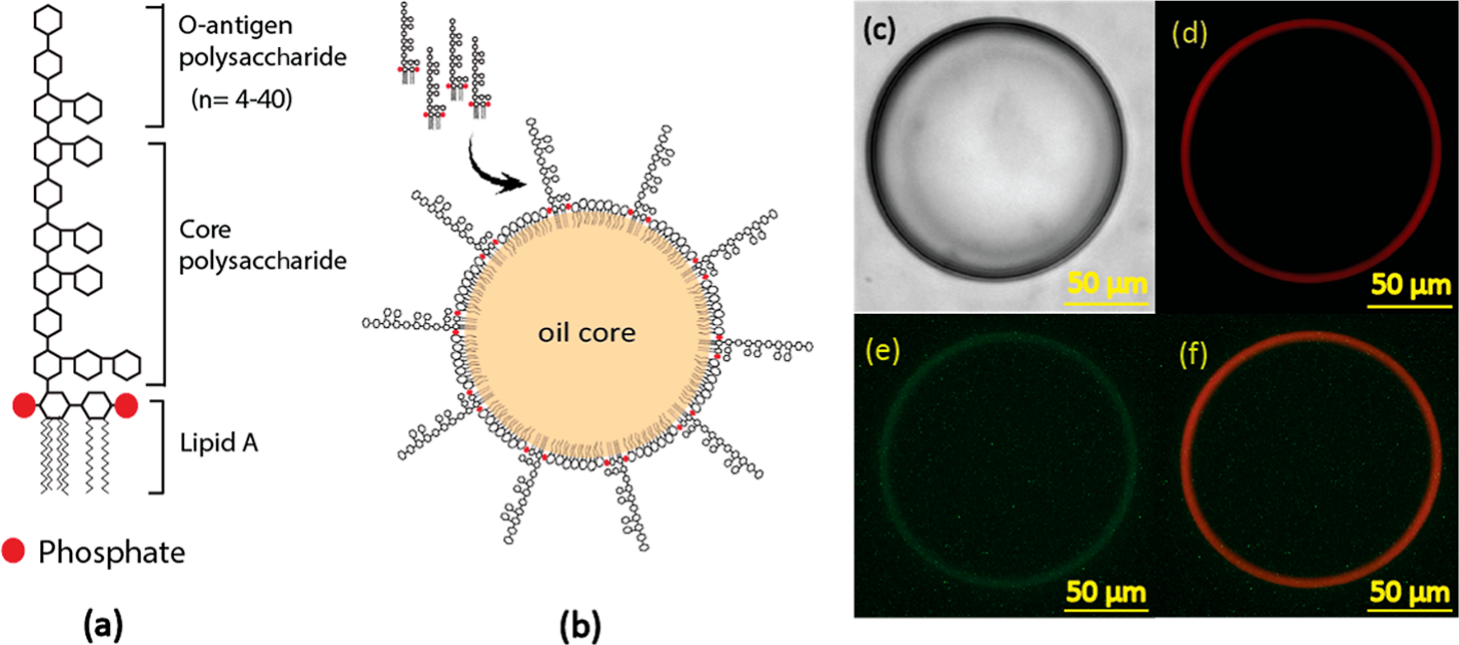
(a) Structure of lipopolysaccharide (LPS), red circles represent negatively charged phosphates linked to lipid A, (b) schematic for the possible LPS adsorption onto lipid droplets, (c) giant lipid droplet under brightfield, (d) epifluorescence microscope image of the lipid droplet labeled with Texas Red-DHPE on the droplet surface, (e) FITC-labeled LPS (FITC-LPS) stays on the surface of the lipid droplet, and the (f) merger of Texas Red and FITC channels.

Lipopolysaccharide (LPS), also known as endotoxin, is a vital component of the outer cell membrane of Gram-negative bacteria.^1^ LPS consists of a hydrophobic lipid moiety, called lipid A, along with a distal polysaccharide known as O-antigen and an oligosaccharide core (Figure 1a). LPS plays an important role in inducing the toxicity elicited by Gram-negative bacteria. LPS binds to a series of pattern recognition receptors, including toll-like receptor 4 (TLR4), and triggers an inflammatory response that produces pro-inflammatory cytokines. Overproduction of proinflammatory molecules can lead to septic shock, which results in fatal clinical consequences. Neutralization of LPS is one of the therapeutic approaches to reduce mortality in septic patients.^2^ For example, hemoperfusion and anti-LPS antibodies have been developed to treat septic shock, but the clinical trials showed inconclusive results.^3,4^

Another LPS neutralization strategy is using natural or synthetic lipoproteins to clear LPS from the bloodstream. It is known that lipoproteins play an important role in clearing endotoxins.^5^ Septic patients typically suffer from lower levels of lipoproteins, which could lead to worse clinical outcomes.^5,6^ Therefore, administrations of natural or synthetic lipoproteins have been used to inhibit endotoxin-induced mortality and restrict LPS activity. Although preclinical studies demonstrated the efficacy of lipoprotein treatment, preclinical and clinical trial results are inconsistent.^7−11^ To maximize therapeutic efficacy, it is critical to identify the key parameters influencing LPS–lipoprotein interactions.

Lipoproteins contain a hydrophobic core surrounded by amphiphilic molecules. The hydrophobic core comprises triglycerides and cholesterol esters, while amphiphilic molecules, including phospholipids, cholesterol, and apolipoproteins, encircle the hydrophobic core.^12^ These lipoproteins are essential for the small intestine’s ability to absorb and transport dietary lipids and move lipids from the liver to peripheral tissues and back to the liver and gut.^13^ The secondary function is to transport harmful foreign hydrophobic and amphipathic substances, including bacterial endotoxins, from invasion and infection sites. Based on their size, lipid content, and apolipoproteins, plasma lipoproteins are classified into chylomicron remnants, chylomicrons, very low-density lipoproteins (LDLs), intermediate-density lipoproteins, and high-density lipoproteins (HDLs). According to recent findings, chylomicrons, LDLs, and HDLs have been shown to increase LPS removal.^14,15^

The uptake of LPS by lipoproteins removes LPS from circulation, and then LPS-attached lipoproteins are broken down in the liver.^13^ Previously, a few physiological parameters impacting LPS clearance have been investigated. For example, a mild LPS-binding protein (LBP) elevation lowers LPS activity.^14^ LBP is an essential carrier delivering the amphiphilic LPS monomer to lymphocyte membranes for TLR4 signaling and lipoproteins for LPS clearance.^16^ Increases in LBP concentrations can assist in LPS clearance and reduce immune responses.^17^ Apoprotein, one of the lipoprotein components, is the other variable that positively impacts the LPS adsorption onto lipoproteins.^14^ Other physicochemical parameters, however, have not yet been investigated.

This study investigated the parameters influencing the adsorption of LPS to synthetic lipid droplets that mimic lipoproteins. The synthetic lipid droplets consist of a triglyceride core surrounded by phospholipids and cholesterol, structurally resembling oil-in-water nanoemulsions. The influences of phospholipids, cholesterol compositions, lipid droplet size, LBP concentration, and bivalent cations on LPS adsorption were investigated. Interestingly, calcium ions significantly impact LPS adsorption near physiological concentrations. This finding suggests that calcium supplementation may improve the efficacy of lipoprotein treatment in septic patients.

## 2 Results

### 2.1 LPS Adsorption on Lipid Droplets

As LPS is an amphiphilic molecule, we hypothesized that LPS would adsorb to the surface of the lipid droplet by embedding its lipid tail in the oil core of the lipid droplet. The hydrophilic polysaccharide chains extend toward the aqueous phase. The LPS uptake process was first observed by fluorescence microscopy. For visualization, giant lipid droplets (80–130 μm in size) labeled with 1,2-dihexadecanoyl-*sn*-glycero-3-phosphoethanolamine (Texas Red-DHPE) were fabricated and then incubated with FITC-labeled LPS (FITC-LPS). Under fluorescent microscopy, we observed LPS majorly adsorbed on the surface of the lipid droplets (Figure 1). To further corroborate the surface adsorption of LPS, the lipid droplet size before and after LPS incubation was measured using dynamic light scattering (DLS) (Figure S1 and Table S1). The increase in the hydrodynamic diameter of lipid droplets was observed after LPS adsorption due to the long polysaccharide chains on the LPS molecules.

### 2.2 Influences of Lipid Droplet Size and Cholesterol

According to the literature, the insertion of amphiphilic molecules into cell membranes is curvature-sensitive.^18^ A high curvature lipid bilayer introduces mismatches to the densely packed phospholipids, thus exposing hydrophobic areas to the aqueous medium as defects.^19^ Such defects offer sites to recruit amphiphilic molecules. Thus, the lipid droplet size may be an important factor for LPS adsorption.

**Figure 2:**
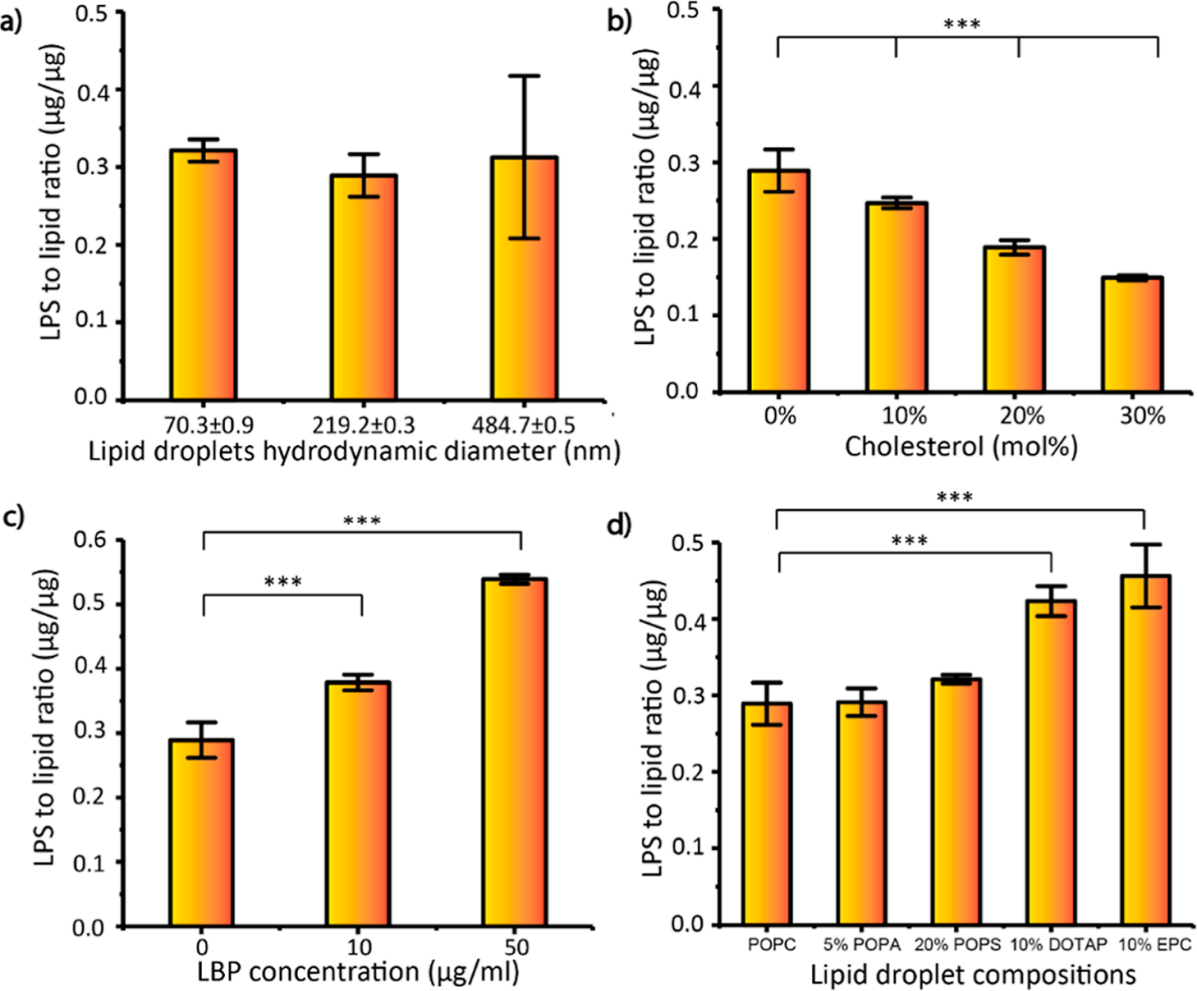
Effect of different parameters on LPS adsorption by lipid droplets. (a) Effect of lipid droplet size, (b) effect of cholesterol, (c) effect of LBP concentration, and (d) effect of charged lipids in the lipid droplet composition. The data were collected from three technical replicates for each run for analysis. ***p value < 0.001 and **p value < 0.01.

The sizes of the lipid droplets were altered by changing lipid-to-triglyceride molar ratios. DLS determined the final lipid droplet sizes after lipid droplet fabrication (Table S1). For the LPS adsorption measurement, the droplets labeled with Texas Red-DHPE were incubated with the FITC-labeled LPS, and the fluorescent signals quantified the amounts of LPS and droplets. Because LPS mainly adsorbs on the surface of the lipid droplets, the total surface area of the lipid droplets would influence the amount of LPS adsorption. Therefore, the LPS uptake efficiency is expressed as the ratios of adsorbed LPS molecules to phospholipids in droplets (Figure 2). For the lipid droplet [molar composition: 99.25% 1-palmitoyl-2-oleoyl-*sn*-glycero-3-phosphocholine (POPC), 0.25% Texas Red-DHPE, and 0.5% biotinyl 1,2-dioleoyl-*sn*-glycero-3-phosphoethanolamine-*N*-(biotinyl) (PE)] in 1x PBS buffer, the LPS to phospholipid ratio is 0.29 ± 0.03 μg/μg. The molecular weight of POPC is 760.076 g/mol. According to the literature, the molecular weight of an LPS monomer ranges from 10 to 20 kDa.^20,21^ Assuming that the molecular weight of LPS is 10 kDa, the LPS to phospholipid molar ratio was around 1:45 (i.e., a single LPS molecule was surrounded by 45 phospholipids).

We prepared lipid droplets in the range of 70–500 nm in diameter. In this size range, we did not observe a significant impact of droplet size on LPS adsorption (Figure 2a). Curvature dependence is also associated with the size of the hydrophobic portions in amphiphilic molecules. Prior studies have reported that the interfacial tension becomes significant for lipid droplets with a radius of ∼5 nm when the cross-sectional area per lipid molecule is lower than ∼1.4 nm.^22^ Because the area per LPS molecule is 1.09 nm^2^,^23^ the curvature may play an important role for smaller lipid droplets.

Human lipoproteins contain free cholesterol or cholesterol ester. Chylomicrons can have up to 1–3 wt % cholesterol ester and free cholesterol,^24^ and HDL contains around 28 wt % cholesterol ester and 6% free cholesterol.^25^ Therefore, we evaluated the influence of cholesterol content by varying cholesterol compositions from 0 to 30 mol %. The increase in the cholesterol composition in lipid droplets reduced the LPS uptake (Figure 2b). The lipid droplets containing 30 mol % cholesterol showed approximately 50% of the LPS uptake compared to the droplets without cholesterol.

### 2.3 Influence of LPS-Binding Proteins and Charged Lipids

LPS-binding protein (LBP) could disassemble LPS aggregates and deliver the LPS monomer to lymphocyte membranes and lipoproteins.^26^ Studies have shown that the amount of LBP present in the blood of septic patients can be used as an important biomarker for the sepsis process.^27^ Clinical studies have reported that the concentration of LBP in the blood is around 4.1 ± 1.65 μg/mL for healthy individuals and ∼31.2 μg/mL (interquartile range, 22.5–47.7 μg/mL) for septic patients.^28^ To observe the influence of LBP, LPS uptake experiments were performed at LBP concentrations ranging from 0 to 50 μg/mL (Figure 2c). A positive correlation between LBP and LPS uptake was observed. The increase in the LBP concentration improves LPS uptake. Compared to 0 μg/mL LBP concentration (control), the LPS adsorption was almost doubled at 50 μg/mL LBP concentration.

The presence of phosphate groups in lipid A gives the net negative charges of LPS.^29^ The electrostatic interactions among LPS, phospholipids, and environmental ions influence the packing density of LPS on lipid droplets. To investigate the influence between charged lipids and LPS, we mixed cationic and anionic phospholipids with neutral POPC in the lipid droplet. We introduced 10 mol % 1,2-dioleoyl-3-trimethylammonium propane (DOTAP) and 10 mol % (1,2-dioleoyl-*sn*-glycero-3-ethylphosphocholine) (EPC) to get positively charged lipid droplets. DLS was used to measure the droplets’ zeta potential (Table S1). The results (Figure 2d) show that the LPS uptake increased by almost 40% compared to the lipid droplets prepared with neutral POPC. The cationic phospholipids may couple with negatively charged LPS, facilitating the insertion of LPS into the droplet surfaces. In contrast, when we mixed 20 mol % 1-palmitoyl-2-oleoyl-*sn*-glycero-3-phospho-*l*-serine (POPS) or 5 mol % 1-palmitoyl-2-oleoyl-*sn*-glycero-3-phosphate (POPA) with POPC in lipid droplets to introduce negatively charged phospholipids on droplet surfaces, the negatively charged phospholipids did not significantly influence the LPS adsorption.

### 2.4 Influence of Bivalent Cations (Ca^2+^ and Mg^2+^)

**Figure 3:**
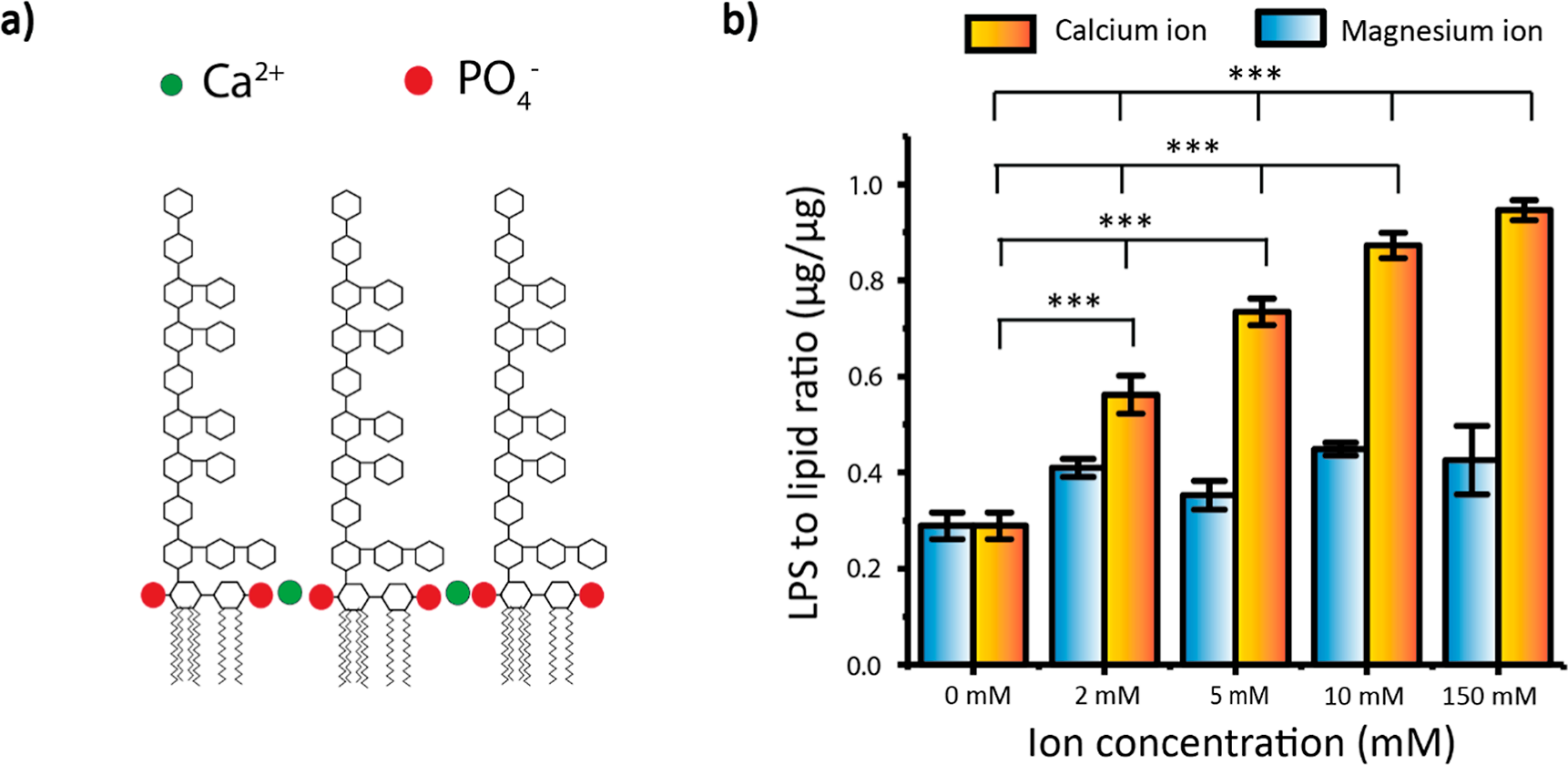
Effect of LPS adsorption by lipid droplets in the presence of bivalent ions in the buffer, (a) mechanism proposed by the literature.^29^ A Ca^2+^ ion stays between LPS molecules, stabilizes negatively charged LPS, and promotes self-insertion of LPS. (b) Effect of different Mg^2+^ and Ca^2+^ concentrations on LPS adsorption. The data were collected from three technical replicates for each run for analysis. ***p value < 0.001 and **p value < 0.01.

Prior studies have shown that the concentration of Ca^2+^ decreases significantly in septic patients.^30−32^ For a healthy population, the median amount of Ca^2+^ ions is around 2.31 mM,^31^ and the Ca^2+^ concentrations in septic patients could be lower than 0.93 mM. In contrast, Mg^2+^ ion concentration does not change much in the blood.^33^ Bivalent cations are known to neutralize and stabilize LPS in the supported lipid bilayers.^29,34^ To observe the effect of bivalent cations in the buffer, Ca^2+^ or Mg^2+^ ions were varied from 0 to 150 mM (Figure 3b). From 2 to 10 mM Ca^2+^ ions, the LPS adsorption nearly doubled, and from 10 to 150 mM, the increase in adsorption was minimal. This increase in LPS adsorption in the presence of the Ca^2+^ ions may be explained by the thermodynamically favorable insertion of LPS in lipid membranes.^29^ The prior study hypothesized that Ca^2+^ ions may bridge negatively charged LPS and promote self-insertion of LPS into a lipid bilayer^29^ (Figure 3a). However, the presence of Mg^2+^ ion in the buffer from 0 to 150 mM did not influence LPS adsorption (Figure 3b).

## 3 Discussion

The nature of LPS uptake by lipoproteins plays an essential role in endotoxin removal. This study used lipid droplets containing phospholipids, triglycerides, and cholesterols but lacking apoproteins to mimic the lipoproteins. Fluorescence microscopy results suggested that the amphiphilic LPS stayed majorly on the surface of lipid droplets (Figure 1). Comparing the size of lipid droplets using DLS, an increase in the droplet size was observed after LPS uptake (Table S1). The DLS data corroborate that LPS is adsorbed on the surface of lipid droplets. The literature suggests that LPS could spontaneously insert into the lipid membrane by burying the hydrophobic tail of LPS in the hydrophobic core of the lipid bilayer.^35^ We hypothesized that LPS also partitions into the phospholipid monolayer on the surface of the lipid droplets by inserting hydrophobic tails into the triglyceride cores. Therefore, the effect of LPS adsorption could be influenced by the droplet curvature, the surface characteristics of lipid droplets, and the local ionic environment.

According to the literature, the insertion of amphiphilic molecules into cell membranes is curvature-sensitive.^18^ Liposomes with high curvatures could introduce defects in phospholipid bilayers, offering recruitment sites for amphiphilic molecules. Thus, we examined the influence of the droplet size on the LPS uptake. However, in our experimental condition for lipid droplets (diameter between 70 and 500 nm), size does not affect LPS adsorption significantly (Figure 2a). Our result is consistent with the other study that evaluated the insertion of fluorescein-labeled DHPE into 50–700 nm diameter liposomes.^19^ The impact of the liposome size on DHPE-fluorescein insertion was insignificant when the liposome size was above 100 nm. For lipoproteins, the curvature dependence of interfacial tension becomes significant when the size of droplets is around 5 nm in radius, if the cross-area of lipid molecules is 1.4 nm^2^.^22^ Because the area per LPS molecule is 1.09 nm^2^,^23^ we expect the curvature may play an important role only in lipid droplets smaller than 10 nm.

The surface molecules of lipid droplets also influence LPS adsorption, including cholesterol and phospholipids. The prior study has shown that cholesterol increases the rigidity of the lipid bilayers and restricts bilayer deformations.^36^ The increase in cholesterol may decelerate the insertion of LPS into lipid droplet surfaces. We observed that the increase in cholesterol contents decreased LPS adsorption, probably due to the restriction of LPS insertion into lipid droplet surfaces. The charged phospholipids also affect LPS adsorption. Due to the negatively charged nature of LPS, the increases of the positively charged lipids, such as EPC and DOTAP, enhance LPS adsorption.

It is known that LBP can dissemble LPS aggregates and deliver the LPS monomer to lipoproteins for LPS clearance.^16,37^ It has been hypothesized that, as a part of the immune response in sepsis, the human body promotes the production of LBP to aid the removal of LPS by lipoprotein, which reduces the amount of LPS in circulation.^38^ The LBP concentration in the blood for healthy individuals is around 4.1 ± 1.65 μg/mL for healthy individuals and ∼31.2 μg/mL (interquartile range, 22.5–47.7 μg/mL) for septic patients.^28^ We also observed that LBP promotes LPS adsorption by lipid droplets. The LPS adsorption increases by ∼40% when LBP concentrations increase from 10 to 50 μg/mL. This implies that LBP could facilitate LPS uptake by lipoprotein. However, LBP can also carry LPS to lymphocyte membranes and trigger TLR4 signaling.^39^ The literature has suggested that LPS neutralization probably depends on the relative amounts of LBPs, lipoproteins, signaling proteins, and target cells.^16^

Cations may also affect the electrostatic interactions of LPS with lipid droplets. We observed that increased Ca^2+^ significantly promotes LPS uptake near the physiological concentration (Figure 3). The literature has proposed that Ca^2+^ ions can bridge LPS molecules, stabilize negatively charged LPS, and promote self-insertion of LPS into lipid layers.^29^ However, the LPS uptake rate was not associated with Mg^2+^ at the same concentrations. A similar phenomenon has been observed in other biomolecular interactions involving carboxylate and phosphate groups.^40−42^ The literature suggests that the stability of hydration shells around cations and the polarization effect are the potential factors influencing the binding affinities of cations.^42−44^ In contrast to Mg^2+^, Ca^2+^ prefers to form direct contact with anions, such as carboxylate/phosphate.^43^ This may explain our observations that Ca^2+^ can enhance LPS uptake by directly binding to negatively charged LPS and bridging them.

The findings from this study can potentially impact the design of treatment strategies for sepsis. Septic shock patients typically suffer from hypocalcemia (lower levels of calcium) and lower levels of lipoproteins. Therefore, calcium or synthetic lipoprotein supplementation has been considered for potential treatment for septic shock patients. However, the preclinical and clinical results are inconclusive.

In the case of calcium supplementation, because the severity and survival rates of septic shock patients were correlated with low Ca^2+^ ion in the blood,^30−32^ calcium replenishment has been recommended to avoid potentially fatal consequences, such as laryngospasm, tetany, seizures, and heart irregularities.^45^ However, neither preclinical nor clinical trials found any benefit of calcium supplements.^46,47^

In the case of lipoprotein supplementation, phospholipid emulsions were administered intravenously to assist in LPS removal. Preclinical studies with injection of endotoxin from *Escherichia coli* followed by the administration of phospholipid emulsions in healthy horses and pigs have shown positive results as the phospholipid emulsion treatment improves survival in sepsis.^9,11^ A double-blind, placebo-controlled clinical trial also showed a positive result with the emulsion treatment. In this clinical study,^8^ before the injection of endotoxin, healthy human volunteers received an infusion of emulsion or placebo over a 6 h period, followed by intravenous administration of endotoxin after 2 h. The volunteers who received emulsion had better clinical outcomes (clinical score, temperature, and pulse rate) and a lower immune response (neutrophil count, tumor necrosis factor α, and interleukin-6). In another large-scale clinical trial, phospholipid emulsion (850 or 1350 mg/kg) and placebo were administered to patients who had confirmed (or were suspected of) Gram-negative sepsis. However, there was no reduction in the 28 day mortality or reduction of new organ failure in patients.^7^

Differences in the Ca^2+^ concentration may explain the discrepancy between preclinical and clinical findings. In the small-scale clinical study, the emulsion was given to healthy individuals before LPS was injected.^8^ Although this study did not report the participants’ Ca^2+^ concentrations, their Ca^2+^ concentrations are likely in the normal range as the study used healthy individuals. Therefore, the supplementation of the emulsion probably assisted in the LPS removal. For the large-scale clinical trial,^7^ the emulsion was given to the septic patients who typically suffer from hypocalcemia;^31^ thus, the low Ca^2+^ concentration in blood may have reduced the efficiency of LPS removal by emulsions. Thus, a combination of calcium and lipid emulsion supplements promises as a better therapeutic approach. Further clinical studies are needed to test the efficacy of this therapeutic approach against sepsis.

## 4 Conclusions

In summary, we demonstrate that physicochemical properties and environmental factors, such as surface charge, surrounding ions, cholesterol levels, and LBP concentrations, control LPS uptake by lipid droplets. Most interestingly, the LPS uptake rate is significantly influenced by Ca^2+^ ions near physiological concentrations. Because septic shock patients typically suffer from hypocalcemia (lower level of calcium) and lower levels of lipoproteins, the supplementation of Ca^2+^ ions along with synthetic lipoproteins may be a potential therapeutic approach for Gram-negative sepsis.

## 5 Methods

### 5.1 Materials

POPC, DOTAP, EPC, POPA (sodium salt), POPS (sodium salt), and biotinyl PE (sodium salt) were sourced from Avanti polar lipids. Cholesterol (3β-hydroxy-5-cholestene, 5-cholesten-3β-ol), glyceryl trioctanoate, LPS-FITC (*E. coli* serotype O55:B5, CDC 1644-70 strain conjugated to fluorescein isothiocyanate), streptavidin, blocker casein, Fisher Bioreagents 10× phosphate-buffered saline (1.37 M sodium chloride, 0.027 M potassium chloride, and 0.119 M phosphates, pH ∼ 7.4), magnesium chloride, calcium chloride, and HEPES (4-(2-hydroxyethyl)-1-piperazineethanesulfonic acid) were purchased from MilliporeSigma. Texas Red-DHPE (triethylammonium salt) and Dynabeads streptavidin T1 were purchased from Fisher Scientific.

### 5.2 Lipid Droplet Preparation

Lipid droplets were synthesized using the ultrasonic emulsification technique.^48^ Phospholipids, Texas Red-DHPE, cholesterol, and biotinylated lipids were dissolved in chloroform and mixed at the desired composition in a 25 mL glass reactor. Chloroform was then evaporated using a rotary evaporator at 40 °C. The lipid thin film was hydrated using 1× PBS solution or HEPES buffer. After hydration, glyceryl trioctanoate (oil phase) was mixed with the aqueous solution to reach a concentration of 2.5% vol/vol. Subsequently, the sample was sonicated for 15 min using a tip sonicator (Qsonica, Q125). The samples were kept in an ice bath during sonication to prevent overheating. After tip sonication, the size and zeta potential of the prepared lipid droplets were characterized and stored for further use. The final total lipid concentration was 5 mg/mL. The continuous phase of the system is an aqueous solution, either 1× PBS buffer or HEPES buffer, depending on the experimental conditions. When comparing the results obtained with HEPES buffer without added calcium ions (Figure 3b) to those with PBS buffer (Figure 2), we observed similar LPS uptake rates. This observation suggests that the anionic buffering molecules in these buffers do not significantly influence LPS uptake by lipid droplets.

### 5.3 Size and Zeta-Potential Measurement

The size and zeta potential of lipid droplets were characterized by using DLS (Malvern Zetasizer Nano ZS90, Malvern Instruments). A serial dilution of 5 mg/mL lipid droplet solution was prepared to minimize multiparticle scattering. The size measurement remained constant, irrespective of the dilution factor. Zeta potential was measured at room temperature by using disposable folded capillary cells. A minimum of three measurements per sample were taken.

### 5.4 LPS Adsorption Measured by Fluorescent Spectroscopy

**Figure 4:**
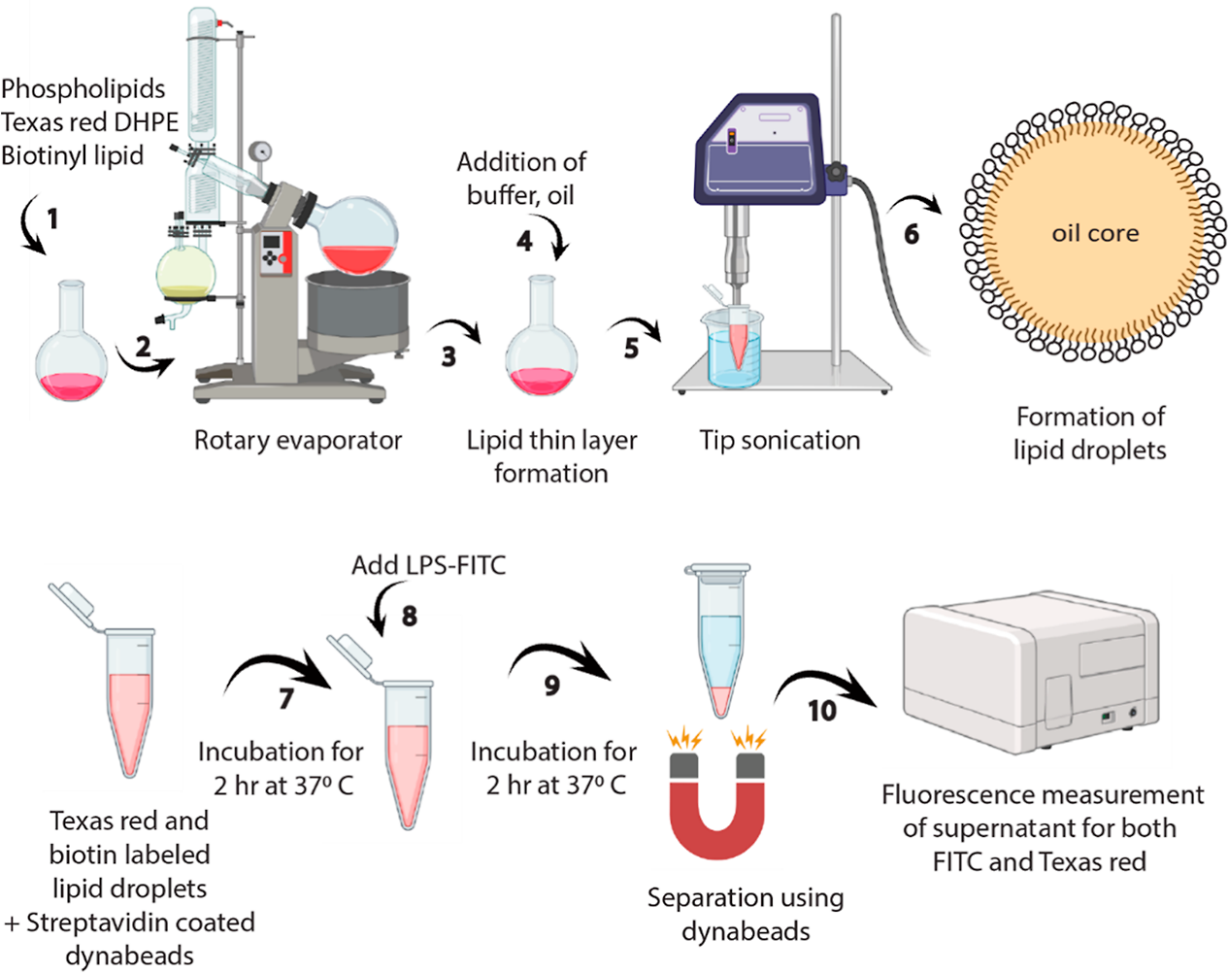
Schematic for lipid droplet preparation and studying their interactions with lipopolysaccharides.

All of the experiments were carried out at a physiological pH of 7.4. For this purpose, 1xPBS buffer was used, except for the study of bivalent ions, where 20 mM HEPES buffer was used. In all of the experiments, the total lipid amount was kept constant. The stock solution of lipid droplets (5 mg/mL total lipid) was diluted 100-fold with the desired buffers. Then, 100 μL of diluted lipid droplets, 24 μL of streptavidin-coated Dynabeads, and the desired buffer volume (1× PBS) with or without LBP were mixed. In the study of bivalent ions, all of the dilutions and pH were maintained using a 20 mM HEPES buffer. CaCl_2_ or MgCl_2_ salt solution in a 20 mM HEPES buffer (final pH 7.4) was added to get the final concentration from 0 to 150 mM. The mixture was incubated at 37 °C for 2 h. Streptavidin on Dynabeads would capture lipid droplets decorated with biotinylated lipids. The lipid droplet solutions were then incubated with 100 μL of 1 mg/mL LPS labeled with the fluorophores (FITC-LPS) for 2 h at 37 °C. Dynabeads were then separated from the solutions using a magnetic rack. The supernatants of lipid droplet-LPS mixtures were collected, and the fluorescence signal was measured using the FLUOstar Omega Microplate Reader (Figure 4).

The fluorescence signals quantified LPS uptake by lipid droplets. Along with the uptake experiments, calibration samples containing the known concentrations of FITC-LPS and Texas Red-labeled droplets were prepared. The amounts of LPS uptake by the lipid droplets that Dynabeads captured were calculated by comparing them with those of the calibration samples. Because the total number of surface lipids would influence the capacity of LPS adsorption, the LPS uptake rates were reported as the mass ratios of adsorbed LPS to lipid molecules.

### 5.5 Imaging LPS Adsorption on Giant Lipid Droplets Using Epifluorescence Microscopy

POPC, Texas Red-DHPE, and biotinylated lipids were mixed at the desired composition in a glass reactor, and chloroform was evaporated by using a rotary evaporator. The lipid thin film was then hydrated using double-deionized (DDI) water. The lipid solution was mixed with glyceryl trioctanoate in a 5:1 volume ratio. The mixture was vortexed for 3 min and diluted 10-fold with DDI water. Giant lipid droplets were then separated from smaller ones using a centrifuge at 1000 RCF for 5 min. A 96-well flat bottom plate coated with streptavidin was prepared for capturing lipid droplets. The well plate with a glass bottom was etched with 1N sulfuric acid, rinsed with DDI water, and then incubated with the streptavidin solution. The well plate was then blocked with a 0.1% casein solution for 1 h. Giant lipid droplets were adsorbed onto glass surfaces via streptavidin–biotin bonding. The unbound droplets were washed with the desired buffer. FITC-LPS was added to the well plate and incubated for 2 h. The excess FITC-LPS was removed by washing the wells. The adsorption of LPS on giant lipid droplets was observed by using a ZEISS Axiovert 200 M fluorescence microscope with a 20× objective. Giant lipid droplets labeled with Texas Red-DHPE were observed by the Texas Red channel (absorption wavelength of 585 nm, emission wavelength of 620 nm), and LPS adsorptions were observed by the FITC channel (absorption wavelength of 485 nm, emission wavelength of 520 nm). The images were processed by using ImageJ software.

## Supporting Information Available

DLS size and zeta-potential measurement of lipid droplets in 1x PBS buffer; molar composition of lipid droplets for characterization and LPS adsorption study; and DLS size measurement (average, *n* = 3) of lipid droplet (99.25% POPC) before and after LPS adsorption at 25 °C for 2 h (PDF)

## Abbreviations

DHPE: 1,2-dihexadecanoyl-*sn*-glycero-3-phosphoethanolamine, triethylammonium salt
HEPES: 4-(2-hydroxyethyl)-1-piperazineethanesulfonic acid
LPS: lipopolysaccharide
LBP: lipopolysaccharide-binding protein
FITC: fluorescein isothiocyanate
FITC-LPS: fluorescein isothiocyanate (FITC)-labeled lipopolysaccharide (LPS)
VLDL: very low-density lipoproteins
LDL: low-density lipoproteins
IDL: intermediate-density lipoproteins
HDL: high-density lipoproteins
POPC: 1-palmitoyl-2-oleoyl-*sn*-glycero-3-phosphocholine
DOTAP: 1,2-dioleoyl-3-trimethylammonium propane
EPC: 1,2-dioleoyl-*sn*-glycero-3-ethylphosphocholine
PA: phosphatidic acid
PS: phosphatidylserine
