# Supplementary material for: Factors Promoting Lipopolysaccharide Uptake by Synthetic Lipid Droplets
Source: ACS Omega. 2025 Feb 10;10(6):5866–73. doi: 10.1021/acsomega.4c09599 (PMC11840781; doi:10.1021/acsomega.4c09599)
Supplement: Supplementary file 1 — ao4c09599_si_001.pdf [file ao4c09599_si_001.pdf]

Supplementary Materials

Factors Promoting Lipopolysaccharide Uptake by Synthetic Lipid Droplets

Assame Arnob[1], Anirudh Gairola[1], Hannah Clayton[1], Arul Jayaraman[1, 2], Hung-Jen Wu[1]*

[1]Artie McFerrin Department of Chemical Engineering, Texas A&M University, College Station, TX 77843, USA
[2]Department of Biomedical Engineering, Texas A&M University, College Station, TX 77843, USA

Table S1: DLS size and zeta potential measurement of lipid droplets in 1x PBS buffer.

Lipid droplet compositions	Before LPS adsorption		After LPS adsorption	
	size	zeta potential	size	zeta potential
99.25 % POPC	211.6 ± 0.9	-7.5 ± 0.5	223.3 ± 2.8	-4.5 ± 0.1
10 % DOTAP + 89.25 % POPC	202.3 ± 0.5	9.3 ± 0.3	213.6 ± 0.1	1.4 ± 0.1
10 % EPC + 89.25 % POPC	196.8 ± .03	6.7 ± 0.2	210.3 ± 0.2	0.0 ± 0.3
20 % POPS + 79.25 % POPC	199.9 ± 0.2	-17.8 ± 0.5	211.5 ± 0.2	-11.1 ± 0.3
5 % POPA + 94.25 % POPC	202.6 ± 0.3	-6.7 ± 0.9	213.5 ± 0.4	-5.0 ± 0.4

Table S2: Molar composition of lipid droplets for characterization and LPS adsorption study.

<table>
<tr><th colspan="2">Study parameter</th><th>POPC (mol %)</th><th>POPA (mol%)</th><th>POPS (mol%)</th><th>DOTAP (mol%)</th><th>EPC (mol%)</th><th>Cholesterol (mol%)</th><th>Texas Red (mol%)</th><th>Biotin (mol%)</th><th>Triglycerides (µl)</th></tr>
<tr><td rowspan="3">Size effect</td><td>70.3 ± 0.9 nm</td><td>99.25</td><td>0</td><td>0</td><td>0</td><td>0</td><td>0</td><td>0.25</td><td>0.5</td><td>2</td></tr>
<tr><td>219.2 ± 0.3 nm</td><td>99.25</td><td>0</td><td>0</td><td>0</td><td>0</td><td>0</td><td>0.25</td><td>0.5</td><td>20</td></tr>
<tr><td>484.7 ± 0.5 nm</td><td>99.25</td><td>0</td><td>0</td><td>0</td><td>0</td><td>0</td><td>0.25</td><td>0.5</td><td>40</td></tr>
<tr><td rowspan="4">Cholesterol effect</td><td>0 mol%</td><td>99.25</td><td>0</td><td>0</td><td>0</td><td>0</td><td>0</td><td>0.25</td><td>0.5</td><td>20</td></tr>
<tr><td>10 mol%</td><td>89.25</td><td>0</td><td>0</td><td>0</td><td>0</td><td>10</td><td>0.25</td><td>0.5</td><td>20</td></tr>
<tr><td>20 mol%</td><td>79.25</td><td>0</td><td>0</td><td>0</td><td>0</td><td>20</td><td>0.25</td><td>0.5</td><td>20</td></tr>
<tr><td>30 mol%</td><td>69.25</td><td>0</td><td>0</td><td>0</td><td>0</td><td>30</td><td>0.25</td><td>0.5</td><td>20</td></tr>
<tr><td rowspan="4">Charge effect</td><td rowspan="2">Positive charge</td><td>89.25</td><td>0</td><td>0</td><td>0</td><td>10</td><td>0</td><td>0.25</td><td>0.5</td><td>20</td></tr>
<tr><td>89.25</td><td>0</td><td>0</td><td>10</td><td>0</td><td>0</td><td>0.25</td><td>0.5</td><td>20</td></tr>
<tr><td rowspan="2">Negative charge</td><td>79.25</td><td>0</td><td>20</td><td>0</td><td>0</td><td>0</td><td>0.25</td><td>0.5</td><td>20</td></tr>
<tr><td>94.25</td><td>5</td><td>0</td><td>0</td><td>0</td><td>0</td><td>0.25</td><td>0.5</td><td>20</td></tr>
</table>

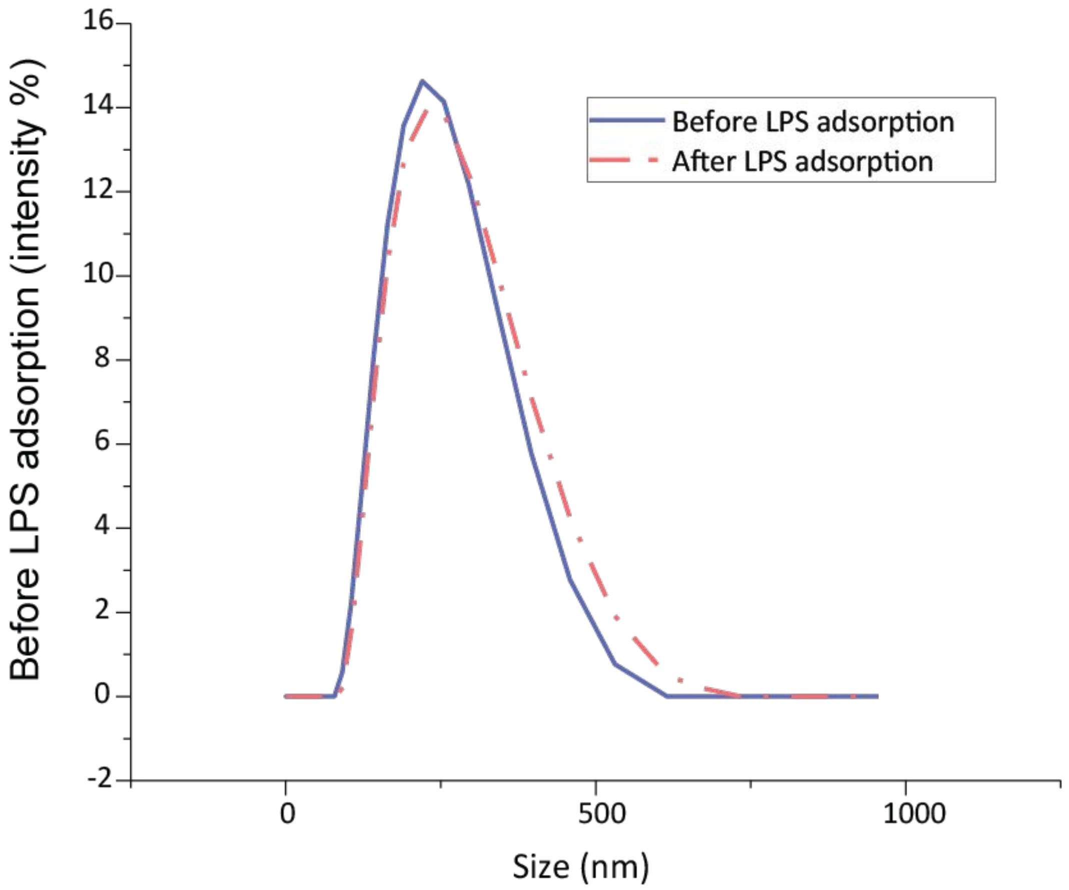

Figure S1. DLS size measurement (average, n=3) of lipid droplet (99.25% POPC) before and after LPS adsorption at 25^0 C for 2hr.
